# Secretion of transthyretin: molecular mechanisms dependent on the endoplasmic reticulum

**DOI:** 10.3389/fphys.2025.1623185

**Published:** 2025-07-01

**Authors:** Jia Meng, Shan-jun Cai

**Affiliations:** ^1^ Department of Ophthalmology, Affiliated Hospital of Zunyi Medical University, Zunyi, China; ^2^ Guizhou Eye Hospital, Zunyi, China; ^3^ Guizhou Branch of National Eye Disease Clinical Research Center, Zunyi, China; ^4^ Special Key Laboratory of Ocular Diseases of Guizhou Province, Zunyi, China

**Keywords:** endoplasmic reticulum, endoplasmic reticulum quality control, unfolded protein response, transthyretin, vitreous amyloidosis

## Abstract

Hereditary transthyretin amyloidosis (ATTRv) results from genetic mutations that destabilize transthyretin (TTR), leading to the formation of extracellular aggregates and amyloid fibrils. A common pathological feature of ATTRv is the capacity of TTR variants to evade endoplasmic reticulum quality control (ERQC) and be secreted, underscoring the critical role of ER regulation in disease pathogenesis. Notably, the TTR Gly83Arg mutation causes familial vitreous amyloidosis, a subtype distinguished by abnormal TTR deposition in the ocular vitreous cavity. Current therapies for ATTRv are ineffective in crossing the blood-retinal barrier or in halting the progression of ocular amyloidosis. This review summarizes the molecular mechanisms of ER-regulated TTR secretion and explores potential causes of ocular amyloid deposition, aiming to provide mechanistic insights into familial vitreous amyloidosis.

## 1 Introduction

Approximately one-third of the human proteome is directed to the ER, where these proteins must first be folded and assembled before being translocated to downstream secretory pathways. ER proteostasis is primarily regulated by the ERQC pathway. This mechanism maintains ER proteostasis by coordinating protein folding and degradation pathways. As proteins enter the ER, folding pathways facilitate their proper folding and assembly, packaging these mature proteins into vesicles for transport to downstream secretory pathways, while misfolded or improperly assembled proteins are selectively retained in the ER and degraded by the ER-associated degradation (ERAD) pathway (Hwang and Qi, 2018; Romine and Wiseman, 2020; Sun and Brodsky, 2019).

Although the ERQC pathway effectively monitors and removes misfolded proteins, certain human diseases, such as hereditary amyloidosis, are caused by structurally abnormal proteins that aggregate to form amyloid fibrils and deposit in tissues. In hereditary amyloidosis, TTR is the most common cause, with over 140 different mutations. TTR is a secretory protein; 90% of it is synthesized and secreted by the liver, while 10% is synthesized by the choroid plexus and retinal pigment epithelium (RPE) cells. It exists as a stable tetramer in circulation, transporting retinol (ROL) and thyroxine (T4) (Adams et al., 2023; Sanguinetti et al., 2022; Si et al., 2021). A central pathological feature of ATTRv is that the TTR variant can be secreted in a non-native tetrameric conformation, which then dissociates into monomers and forms amyloid fibrils. Furthermore, different variants display distinct tissue-selective deposition patterns and associated pathologies (Magalhães et al., 2021; Sanguinetti et al., 2022). Among these, the TTR Gly83Arg mutation represents a unique TTR variant recently identified in the Chinese population (including our team’s preliminary work). All these patients exhibited ocular involvement, primarily vitreous amyloidosis. Commonly referred to as familial vitreous amyloidosis (He et al., 2022; Li et al., 2022; Liu et al., 2014; Xie et al., 2017; Yin et al., 2014).

The release of amyloid proteins from tissues is a key driver in the pathogenesis of ATTRv, and the endoplasmic reticulum (ER) plays a crucial regulatory role in this process. In this review, we summarize the mechanisms of ER-regulated TTR secretion and further explore the pathological process of vitreous amyloid deposition.

## 2 TTR secretion is determined by the activity of the endoplasmic reticulum quality control pathway

ATTRv is caused by mutations in the TTR gene that disrupt its native conformation, leading to the misfolding of the protein and the eventual formation of amyloid fibrils. The ERQC regulates TTR folding, trafficking, and degradation, and various TTR variants may undergo differential regulation, resulting in tissue-specific deposition patterns. Thus, the activity of ERQC pathways that mediate TTR secretion determines its output. Within this control framework, two main factors influence the secretion of proteins into the extracellular compartment (Chen et al., 2015).

**FIGURE 1:**
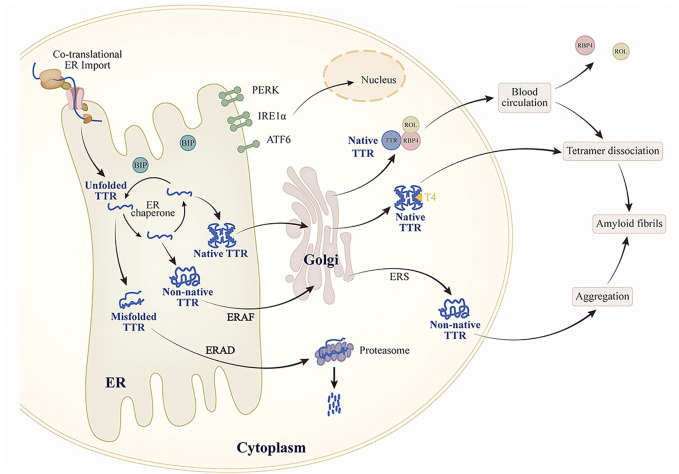
Schematic of TTR secretion and extracellular aggregation.

One of the factors is the intrinsic energetic stability of protein folding, which includes thermodynamic stability (the tendency to acquire the folded conformation) and kinetic stability (the folding rate). The energetic stability of a protein determines its ability to adopt a folded conformation in ER homeostasis. This connection between protein stability and protein secretion has been confirmed in some TTR variants studies. Studies have found that patients with the highly amyloidogenic and unstable TTR variant (TTR D18G) do not show severe systemic pathological manifestations, presenting only with late-onset central nervous system disorders. Further cellular experiments revealed that in cells lacking endogenous TTR expression, TTR D18G is recognized and degraded by ERQC, reducing its secretion and extracellular aggregation. In contrast, the highly amyloidogenic but moderately unstable TTR L55P variant can escape ERQC as a tetramer, with secretion levels comparable to those of the stable wild-type TTR. This characteristic results in early-onset ATTRv, the most aggressive form in patients carrying TTR L55P (Chen et al., 2015; Frangolho et al., 2020; Sekijima et al., 2005; Sörgjerd et al., 2006). This implies that unstable TTR variants can still fold in the ER to form stable conformations. Furthermore, T4 and small molecules targeting the T4-binding pocket may also enhance the stability of TTR variants. TTR, in its native tetramer form, has two hydrophobic pockets bound to T4. However, only one binding site can attach to T4 (Yin et al., 2014). The choroid plexus may contain large amounts of T4 and lack competitive T4-binding proteins (Dickson et al., 1987). In the rat choroid plexus cells, TTR A25T is secreted into the cerebrospinal fluid (CSF) as efficiently as wild-type TTR, and the addition of T4 enhances this secretion. This indicates that T4 stabilizes the TTR variant, allowing it to escape ERQC and be secreted into the CSF. However, the relatively low levels of T4 in the CSF are insufficient to maintain the stability of the TTR variant, ultimately leading to its dissociation (Hammarström et al., 2003; Sekijima et al., 2003). Although T4 is also found in the liver, high-affinity T4-binding proteins in the liver competitively bind T4, thus reducing the amount of T4 available to stabilize TTR variants (Hamilton and Benson, 2001; Sekijima et al., 2005; Yin et al., 2014) (Figure 1).

Another factor is the activity of the protein folding and degradation pathways in the ER, both of which influence the non-native conformation of proteins. The balance between ER-assisted folding (ERAF) and ERAD pathways significantly affects the efficiency of TTR secretion (Sekijima et al., 2005; Wiseman et al., 2007). Thus, while the ERQC system can recognize unstable TTR variants and degrade them via the ERAD pathway, ERAD cannot prevent the secretion of TTR variants capable of forming tetramers; these tetramers can be secreted through the ERAF pathway. For example, in transiently transfected cells that do not express endogenous TTR, ERQC captures and prevents the secretion of monomeric forms of stable, early-onset TTR variants (such as TTR V30M) but allows the secretion of their tetrameric forms (Sato et al., 2007). Different tissues collectively influence protein secretion by regulating their ER protein folding, translocation, and degradation pathways. This regulation adapts to tissue properties, environmental conditions, or metabolic demands. The effect is mediated by the unfolded protein response (UPR) (Figure 1).

## 3 Regulation of TTR by the unfolded protein response

**FIGURE 2:**
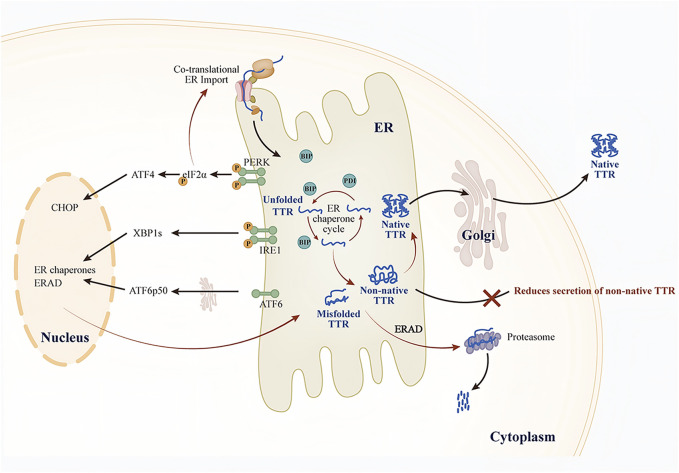
Effect of UPR on TTR secretion.

The UPR comprises three key endoplasmic reticulum transmembrane proteins: protein kinase R-like ER kinase (PERK), inositol-requiring enzyme 1 (IRE1), and activating transcription factor 6 (ATF6) (Karagöz et al., 2019). When misfolded proteins accumulate and induce endoplasmic reticulum stress (ERS), BiP preferentially binds to these proteins, thus promoting the IRE1, PERK, and ATF6 signaling pathways (Figure 2). In the early stages of the UPR, adaptive reorganization of endoplasmic reticulum homeostasis occurs, enhancing cellular physiological functions. This remodeling can ease ERS and restore the homeostasis of the ER protein folds. However, when chronic or severe ER damage takes place, the PERK and IRE1 signaling pathways suppress adaptive responses and initiate apoptosis (Iurlaro and Muñoz-Pinedo, 2016; Hetz et al., 2020; Preissler and Ron, 2019). Here, we focus solely on the role of the UPR in regulating TTR secretion and extracellular aggregation.

Ensuring the activity of ERQC pathways is essential for maintaining ER proteostasis. Consequently, dysregulation of ERQC pathways in target tissues (e.g., ERS) may disrupt ER proteostasis and contribute to the development of amyloidosis (Romine and Wiseman, 2020). The conventional view holds that the primary function of ER proteostasis is to prevent the secretion of misfolded or non-native conformational proteins. However, the secretion of such aberrant proteins may serve as a protective mechanism to lessen the burden of ERS. Specifically, the secretion of non-native conformation TTR variants may represent a compensatory mechanism initiated by cells to restore ER proteostasis during ERS. For example, small-molecule fluorogenic TTR ligands emit fluorescence upon binding to and forming covalent linkages with TTR tetramers. Using these molecules, researchers discovered that unstable TTR variants (such as TTR A25T) can be secreted both in their native tetramers and in non-native conformations. In a mammalian cell culture model, although thapsigargin (Tg) induced ERS, the total TTR A25T decreased while TTR aggregates in the cell culture medium increased (Chen et al., 2016). Notably, Tg-induced ERS promoted the secretion of TTR in non-native tetrameric conformations, and these aggregates are typically closely associated with distal toxicity in the pathogenesis of TTR amyloidosis. This ERS-dependent increase in the secretion of non-native TTR explains why the dysregulation of ERS markers in the liver promotes TTR aggregate deposition. This mechanism is further supported by observations in domino liver transplants from ATTRv donors, where recipients show accelerated TTR amyloid deposition (Lladó et al., 2010). ERS can disrupt ER proteostasis. The imbalance in ER proteostasis alters the conformational integrity of TTR and promotes its secretion, ultimately leading to the formation of extracellular amyloid fibrils.

To counteract ERS, cells primarily activate the UPR to remodel ERQC, thereby maintaining ER proteostasis and ensuring the proper folding of TTR while effectively preventing the abnormal secretion and extracellular aggregation of misfolded TTR. (Wiseman et al., 2022) (Figure 2). Research shows that the ATF6 signaling pathway can preferentially reduce the secretion of unstable and aggregation-prone TTR variants. In cell culture models, activation of ATF6 significantly decreased the secretion of TTR aggregates and their subsequent accumulation, even independently of ERS (Chen et al., 2014). Further studies monitoring tetramers, aggregates, and total TTR in the conditioned medium of cells revealed that ATF6 activation did not alter the conformation of TTR secreted by mammalian cells. Instead, it enhanced the interaction of unstable TTR with ER chaperones such as BiP and PDIA4, promoting the retention of unstable TTR in the ER and thereby reducing the total amount of secreted protein, ultimately lowering the levels of TTR aggregates in the conditioned medium. In contrast, Tg-induced ERS reduced TTR tetramers in the conditioned medium but increased the secretion of TTR aggregates. Additionally, the study uncovered the synergistic role of ATF6-regulated BiP and PDIA4 in modulating TTR secretion; however, the regulatory effects varied across cell types. For example, PDIA4 reduced the secretion of unstable TTR variants in human embryonic kidney 293T cells (HEK293T) and human hepatocellular carcinoma cells (HepG2), whereas BiP overexpression exhibited a similar effect only in HEK293T cells (Mesgarzadeh et al., 2022). Similarly, the XBP1 signaling pathway is also involved in regulating the folding, transport, and degradation of unstable, aggregation-prone proteins through a mechanism similar to that of the ATF6 signaling pathway (Romine and Wiseman, 2020; Shoulders et al., 2013).

In contrast to the ATF6 and IRE1/XBP1 pathways, the PERK signaling pathway is regulated through both transcriptional and translational mechanisms during ERS. PERK activation induces translational attenuation, which reduces the co-translational influx of newly synthesized proteins into the endoplasmic reticulum. The study found that in mammalian cells, compared to treatment with Tg alone, the combined treatment with a PERK inhibitor and Tg not only increased the secretion of total TTR A25T but also altered its conformational distribution: the secretion of the native tetrameric form decreased, while the non-native conformations (mainly existing as soluble oligomers) increased. Similarly, the conformation of the stable wild-type TTR was also affected by PERK. This suggests that the PERK signaling pathway plays a crucial role in determining extracellular proteostasis by regulating the conformational integrity of TTR (Romine and Wiseman, 2019). Since secretory proteostasis depends on the UPR, dysregulation of the UPR in cells that produce pathological amyloidogenic proteins may inadvertently make the extracellular environment more vulnerable to ER stress-mediated toxic protein aggregation. Therefore, remodeling ER proteostasis can effectively decrease the secretion and extracellular aggregation of TTR variants without impacting wild-type TTR secretion (Plate and Wiseman, 2017). Targeting UPR-dependent ER regulation, especially the ATF6 signaling pathway, offers a novel strategy to reduce the secretion and toxic aggregation of proteins linked to ATTRv pathology.

## 4 TTR secretion mechanism in vitreous amyloidosis and research prospects

In patients with ATTRv, ocular involvement typically occurs in the later stages of the disease, with clinical manifestations including vitreous opacities, chronic open-angle glaucoma, abnormal conjunctival vessels, and keratoconjunctivitis sicca, among others (Minnella et al., 2021). Notably, ocular manifestations vary significantly depending on the specific TTR mutation, and even the same mutation site may exhibit inconsistent phenotypic characteristics across different regional studies (Reynolds et al., 2017). We have listed the TTR mutations associated with vitreous amyloidosis (Table 1). In patients with familial vitreous amyloidosis (e.g., those carrying the TTR Gly83Arg mutation), vitreous opacities are typically the initial symptom, and ocular symptoms usually appear earlier than neurological symptoms. Our recently published study on the long-term follow-up of vitreous amyloid deposition caused by the TTR Gly83Arg mutation demonstrated a 100% incidence of vitreous opacity in mutation carriers, and patients experience recurrence after vitrectomy (Chen et al., 2025). Vitreous biopsy specimens from TTR Gly83Arg patients showed prominent amyloid deposits on Congo red staining, with immunohistochemistry confirming TTR amyloid deposition (Liu et al., 2014). Furthermore, while liver transplant recipients exhibited a significant reduction in serum levels of the TTR variant, their ocular manifestations did not improve markedly (Hara et al., 2010). In addition to hepatocytes, RPE can also synthesize and secrete TTR. Therefore, the TTR amyloid deposits in the vitreous cavity are not derived from the liver but are likely produced by RPE cells. Current therapeutic strategies targeting TTR synthesis, secretion, and extracellular aggregation, such as liver transplantation, TTR gene silencers (including RNA interference therapies [Patisiran and Vutrisiran] and antisense oligonucleotides [Inotersen]), and TTR stabilizers (including Tafamidis, Difunisal, and Acoramidis) are only applicable for treating ATTRv polyneuropathy or ATTRv cardiomyopathy (Adams et al., 2023; Ando et al., 2022). Although trace amounts of tafamidis have been found in the cerebrospinal fluid and vitreous humor of treated patients, it has not been conclusively proven that tafamidis effectively crosses the blood-brain barrier or blood-retinal barrier (Monteiro et al., 2018). Our previous study showed that vitrectomy provides temporary visual improvement but does not stop the ongoing secretion and deposition of TTR variants. To date, no clinical evidence has confirmed that any approved or novel therapies can effectively delay the progression of ocular symptoms, likely due to their inability to penetrate the blood-retinal barrier.

**TABLE 1:** Mutations associated with ocular involvement (www.amyloidosismutations.com).

Mutation	Early or classic symptom(s)	Reference
Cys10Arg	polyneuropathy, vitreous opacities, cardiomyopathy	Uemichi et al. (1992)
Ser23Asn	Cardiomyopathy, vitreous opacities	Connors et al. (1999)
Val30Met	Polyneuropathy, vitreous opacities	Ishida et al. (2017)
Val30Gly	central nervous system, vitreous opacities	Martin et al. (2014)
Phe33Cys	vitreous opacities, cardiomyopathy	Lim et al. (2003)
Phe33Ile	vitreous opacities, polyneuropathy	Jacobson et al. (1988)
Arg34Gly	vitreous opacities	Levy et al. (2012)
Lys35Thr	vitreous opacities	Long et al. (2012)
Ala36Pro	polyneuropathy, vitreous opacities	Jones et al. (1991)
Trp41Leu	vitreous opacities	Yazaki et al. (2002)
Gly53Ala	polyneuropathy, vitreous opacities, cardiomyopathy	Douglass et al. (2007)
Glu54Gly	polyneuropathy, vitreous opacities	Reilly et al. (1995)
Glu54Lys	polyneuropathy, vitreous opacities	Togashi et al. (1999)
Leu55Gln	Glaucoma, vitreous opacities, polyneuropathy	Yazaki et al. (2002)
Leu55Arg	vitreous opacities, polyneuropathy	Long et al. (2012)
Leu55Pro	polyneuropathy, vitreous opacities	Jacobson et al. (1992)
Leu58Arg	carpal tunnel syndrome, vitreous opacities	Saeki et al. (1991)
Phe64Ser	vitreous opacities, polyneuropathy	Uemichi et al. (1999)
Tyr69His	polyneuropathy, vitreous opacities	Schweitzer et al. (2009)
Lys70Asn	carpal tunnel syndrome, vitreous opacities	Izumoto et al. (1992)
Val71Ala	carpal tunnel syndrome, vitreous opacities	Almeida Mdo et al. (1993)
Gly83Arg	vitreous opacities	Xie et al. (2017)
Ile84Asn	vitreous opacities, carpal tunnel syndrome, cardiomyopathy	Skinner et al. (1992)
Ile84Ser	carpal tunnel syndrome, vitreous opacities, cardiomyopathy	Dwulet and Benson (1986)
Ala97Ser	polyneuropathy, cardiomyopathy, vitreous opacities	Tachibana et al. (1999)
Tyr114Cys	polyneuropathy, vitreous opacities	Ueno et al. (1990)

TTR G83R represents a distinct mutation type that can induce vitreous amyloidosis, although the precise molecular mechanisms underlying its amyloid fibril formation remain incompletely understood. Based on current research, we have analyzed several potential pathogenic mechanisms: First, as previously discussed in this study, the ER proteostasis regulatory pathway can influence TTR secretion and extracellular aggregation through multiple mechanisms. Compared to other TTR variants, TTR G83R may more easily escape the ERQC system, consequently leading to amyloid deposition in the vitreous cavity.

Second, the vitreous may have a specific affinity for the TTR G83R mutant protein. The structure of TTR indicates that residues K80, L82, G83, and I84 are responsible for forming the EF helical loops of the two subunits of the tetramer, and this region mediates the interactions between the proteins (Ferguson et al., 2021; Zanotti et al., 2008). Research shows that the G83R mutation in TTR brings this subunit closer to the R62 residue of RBP, significantly reducing the stability of the TTR-RBP complex due to electrostatic repulsion, as both share the same charge (Liu et al., 2014). The mutation replaces the neutral hydrophilic glycine with a positively charged arginine at this position, likely enhancing its anion-binding capacity. We know that the vitreous is rich in hyaluronic acid, a polyanionic polymer synthesized and secreted by vitreous cells (Bishop, 2000). Therefore, hyaluronic acid may adsorb TTR G83R, which subsequently aggregates in the vitreous cavity to form amyloid deposits.

**FIGURE 3:**
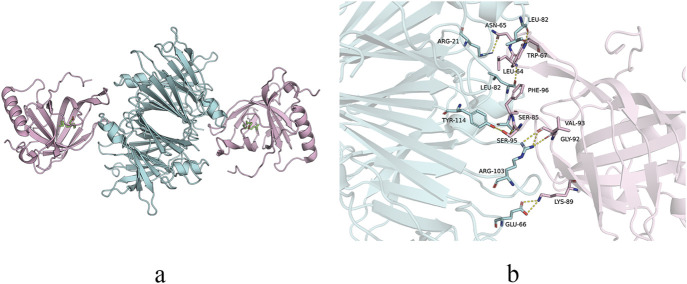
Structure of TTR-RBP complex. **(a)** Structure representation of the TTR-RBP complex. TTR: blue. RBP4: pink. Retinol: green. **(b)** Detail of the contact at the residue level between TTR and RBP4.

Third, under normal physiological conditions, ROL absorbed from dietary sources is stored as retinyl palmitate in hepatic stellate cells (Martin Ask et al., 2021). When needed, ROL is released from retinyl palmitate through hydrolysis by retinyl ester hydrolase (Haemmerle and Lass, 2019; Wagner et al., 2020). The ROL is then transported from stellate cells to hepatocytes via retinol-binding protein 1 on the surfaces of both cell types. Within hepatocytes, ROL binds to retinol-binding protein 4 (RBP4), forming the holo-RBP4 complex, which subsequently associates with TTR to create the ternary holo-RBP4-TTR complex (Figure 3). This complex is then secreted from hepatocytes into systemic circulation. The holo-RBP4-TTR complex delivers ROL to RPE cells, where it activates the signaling receptor and transporter of retinol 6 on the cell surface (Kawaguchi et al., 2007). After ROL enters RPE cells to participate in the visual cycle, TTR and RBP4 return to systemic circulation for metabolism by the liver and kidneys (Steinhoff et al., 2022; Yin et al., 2014). Notably, TTR exhibits a very low affinity for RBP4 without ROL. Upon delivery of ROL to the RPE by the holo-RBP4-TTR complex, TTR dissociates from RBP4. *In vitro* studies demonstrate that holo-RBP4 binds to TTR in a concentration-dependent manner to form a complex, which not only stabilizes the TTR tetramer but also prevents its dissociation into selectively folded monomers that are prone to fibril formation. More importantly, in the presence of holo-RBP4, T4 exhibits a stronger inhibitory effect on fibril formation compared to using either T4 or holo-RBP4 alone (White and Kelly, 2001; Yin et al., 2014). Furthermore, when the liver cannot provide enough ROL, other organs can utilize circulating dietary ROL (Nishimoto et al., 2020). A study demonstrated that retinol binding protein receptor 2 (RBPR2) knockout mice supplemented with dietary ROL have decreased retinoid levels in the eye without pathological changes, while RBPR2 knockout mice not supplemented with ROL develop thinning of the photoreceptor layer, resulting in visual impairment (Radhakrishnan et al., 2022). The ROL in the retina is primarily derived from the holo-RBP4-TTR complex delivered to RPE cells via systemic circulation, while a minor portion originates from dietary ROL that enters the retina directly through the retinal capillary network. RPE cells may compensate by secreting TTR to facilitate the transport of this portion of ROL, and subsequent ROL release may predispose TTR to aggregation.

In summary, there is a significant lack of precise treatment options for vitreous amyloidosis. Future research should focus on clarifying its pathogenesis, particularly exploring the regulatory mechanisms of RPE cells that can produce TTR in the eye. A key scientific question is whether specific regulatory factors exist in RPE cells that can influence the conformational stability of TTR variants and mediate their escape from the ERQC system. Additionally, in our TTR Gly83Arg mutant mouse model, vitreous opacity was the sole pathological manifestation, with amyloid deposition detected exclusively in the vitreous while showing negative results in the heart, liver, brain, and kidneys (Ran et al., 2018). This phenomenon provides important support for the compensatory pathological mechanism of the “liver-RPE axis,” whereby gene mutations lead to insufficient TTR secretion by the liver, triggering negative feedback regulation that induces compensatory TTR secretion from the RPE to deliver dietary ROL and maintain visual cycle function, and the subsequent release of ROL may predispose TTR to aggregation. Currently, the mechanism by which RPE cells regulate TTR secretion is a key research priority for our team.

## 5 Conclusion

The ERQC pathway can recognize and retain unstable TTR variants to prevent their secretion. However, any factor that enhances the stability of TTR variants in the ER or activates the ER secretory pathway may allow TTR variants to escape from the ERQC. These secreted non-native tetramers dissociate into monomers, which then aggregate to form amyloid fibrils that ultimately deposit in tissues and organs.

In familial vitreous amyloidosis, ocular involvement typically appears as the first symptom. Importantly, the progression of ocular amyloidosis is unaffected by liver transplantation, potentially because the RPE continues to produce TTR variants locally. Therefore, future research needs to investigate how ER regulates the secretion and extracellular aggregation of TTR variants in different tissues. Elucidating these mechanisms may help clarify the tissue-specific causes of vitreous amyloidosis. Through such efforts, we aim to identify specific biomarkers for monitoring disease progression and guiding targeted therapeutic interventions in vitreous amyloidosis.

## References

[B1] Adams D. Sekijima Y. Conceição I. Waddington-Cruz M. Polydefkis M. Echaniz-Laguna A. (2023). Hereditary transthyretin amyloid neuropathies: advances in pathophysiology, biomarkers, and treatment. Lancet Neurol. 22 (11), 1061–1074. 10.1016/S1474-4422(23)00334-4 37863593

[B2] Adams D. Tournev I. L. Taylor M. S. Coelho T. Planté-Bordeneuve V. Berk J. L. (2023). Efficacy and safety of vutrisiran for patients with hereditary transthyretin-mediated amyloidosis with polyneuropathy: a randomized clinical trial. Amyloid 30 (1), 1–9. 10.1080/13506129.2022.2091985 35875890

[B3] Almeida Mdo R. Lopez-Andreu F. Munar-Qués M. Costa P. P. Saraiva M. J. (1993). Transthyretin ALA 71: a new transthyretin variant in a Spanish family with familial amyloidotic polyneuropathy. Hum. Mutat. 2 (5), 420–421. 10.1002/humu.1380020516 8257997

[B4] Ando Y. Adams D. Benson M. D. Berk J. L. Planté-Bordeneuve V. Coelho T. (2022). Guidelines and new directions in the therapy and monitoring of ATTRv amyloidosis. Amyloid 29 (3), 143–155. 10.1080/13506129.2022.2052838 35652823

[B5] Bishop P. N. (2000). Structural macromolecules and supramolecular organisation of the vitreous gel. Prog. Retin Eye Res. 19 (3), 323–344. 10.1016/s1350-9462(99)00016-6 10749380

[B6] Chen J. J. Genereux J. C. Qu S. Hulleman J. D. Shoulders M. D. Wiseman R. L. (2014). ATF6 activation reduces the secretion and extracellular aggregation of destabilized variants of an amyloidogenic protein. Chem. Biol. 21 (11), 1564–1574. 10.1016/j.chembiol.2014.09.009 25444553 PMC4254654

[B7] Chen J. J. Genereux J. C. Suh E. H. Vartabedian V. F. Rius B. Qu S. (2016). Endoplasmic reticulum proteostasis influences the oligomeric state of an amyloidogenic protein secreted from mammalian cells. Cell Chem. Biol. 23 (10), 1282–1293. 10.1016/j.chembiol.2016.09.001 27720586 PMC5108364

[B8] Chen J. J. Genereux J. C. Wiseman R. L. (2015). Endoplasmic reticulum quality control and systemic amyloid disease: impacting protein stability from the inside out. IUBMB Life 67 (6), 404–413. 10.1002/iub.1386 26018985 PMC4485578

[B9] Chen X. Xin C. Su G. Xie B. Li H. Ren H. (2025). Hereditary vitreoretinal amyloidosis with transthyretin Gly83Arg variant, a long-term study. Eye (Lond). 39 (2), 345–353. 10.1038/s41433-024-03445-y 39478196 PMC11750974

[B10] Connors L. H. Théberge R. Skare J. Costello C. E. Falk R. H. Skinner M. (1999). A new transthyretin variant (Ser23Asn) associated with familial amyloidosis in a Portuguese patient. Amyloid 6 (2), 114–118. 10.3109/13506129909007311 10439117

[B11] Dickson P. W. Aldred A. R. Menting J. G. Marley P. D. Sawyer W. H. Schreiber G. (1987). Thyroxine transport in choroid plexus. J. Biol. Chem. 262 (29), 13907–13915. 10.1016/s0021-9258(18)47880-5 3654646

[B12] Douglass C. Suvarna K. Reilly M. M. Hawkins P. N. Hadjivassiliou M. (2007). A novel amyloidogenic transthyretin variant, Gly53Ala, associated with intermittent headaches and ataxia. J. Neurol. Neurosurg. Psychiatry 78 (2), 193–195. 10.1136/jnnp.2006.093500 16971399 PMC2077663

[B13] Dwulet F. E. Benson M. D. (1986). Characterization of a transthyretin (prealbumin) variant associated with familial amyloidotic polyneuropathy type II (Indiana/Swiss). J. Clin. Invest 78 (4), 880–886. 10.1172/JCI112675 3760189 PMC423707

[B14] Ferguson J. A. Sun X. Dyson H. J. Wright P. E. (2021). Thermodynamic stability and aggregation kinetics of EF helix and EF loop variants of transthyretin. Biochemistry 60 (10), 756–764. 10.1021/acs.biochem.1c00073 33645214 PMC8023301

[B15] Ferreira N. Saraiva M. J. Almeida M. R. (2019). Uncovering the neuroprotective mechanisms of curcumin on transthyretin amyloidosis. Int. J. Mol. Sci. 20 (6), 1287. 10.3390/ijms20061287 30875761 PMC6471102

[B16] Frangolho A. Correia B. E. Vaz D. C. Almeida Z. L. Brito R. M. M. (2020). Oligomerization profile of human transthyretin variants with distinct amyloidogenicity. Molecules 25 (23), 5698. 10.3390/molecules25235698 33287192 PMC7730986

[B17] Haemmerle G. Lass A. (2019). Genetically modified mouse models to study hepatic neutral lipid mobilization. Biochim. Biophys. Acta Mol. Basis Dis. 1865 (5), 879–894. 10.1016/j.bbadis.2018.06.001 29883718 PMC6887554

[B18] Hamilton J. A. Benson M. D. (2001). Transthyretin: a review from a structural perspective. Cell Mol. Life Sci. 58 (10), 1491–1521. 10.1007/PL00000791 11693529 PMC11337270

[B19] Hammarström P. Sekijima Y. White J. T. Wiseman R. L. Lim A. Costello C. E. (2003). D18G transthyretin is monomeric, aggregation prone, and not detectable in plasma and cerebrospinal fluid: a prescription for central nervous system amyloidosis? Biochemistry 42 (22), 6656–6663. 10.1021/bi027319b 12779320

[B20] Hara R. Kawaji T. Ando E. Ohya Y. Ando Y. Tanihara H. (2010). Impact of liver transplantation on transthyretin-related ocular amyloidosis in Japanese patients. Arch. Ophthalmol. 128 (2), 206–210. 10.1001/archophthalmol.2009.390 20142544

[B21] He X. Tian Z. Guan H. Zhang S. (2022). Clinical phenotypes and genetic features of hereditary transthyretin amyloidosis patients in China. Orphanet J. Rare Dis. 17 (1), 337. 10.1186/s13023-022-02481-9 36056432 PMC9438301

[B22] Hetz C. Zhang K. Kaufman R. J. (2020). Mechanisms, regulation, and functions of the unfolded protein response. Nat. Rev. Mol. Cell Biol. 21 (8), 421–438. 10.1038/s41580-020-0250-z 32457508 PMC8867924

[B23] Hetz C. Papa F. R. (2018). The unfolded protein response and cell fate control. Mol. Cell 69 (2), 169–181. 10.1016/j.molcel.2017.06.017 29107536

[B24] Hwang J. Qi L. (2018). Quality control in the endoplasmic reticulum: crosstalk between ERAD and UPR pathways. Trends Biochem. Sci. 43 (8), 593–605. 10.1016/j.tibs.2018.06.005 30056836 PMC6327314

[B25] Ishida K. Nishida T. Niimi Y. Suemori S. Mochizuki K. Kawakami H. (2017). Elderly onset vitreous opacities as the initial manifestation in hereditary transthyretin (ATTR Val30Met) carries. Ophthalmic Genet. 38 (4), 387–391. 10.1080/13816810.2016.1232413 28085522

[B26] Iurlaro R. Muñoz-Pinedo C. (2016). Cell death induced by endoplasmic reticulum stress. FEBS J. 283 (14), 2640–2652. 10.1111/febs.13598 26587781

[B27] Izumoto S. Younger D. Hays A. P. Martone R. L. Smith R. T. Herbert J. (1992). Familial amyloidotic polyneuropathy presenting with carpal tunnel syndrome and a new transthyretin mutation, asparagine 70. Neurology 42 (11), 2094–2102. 10.1212/wnl.42.11.2094 1436517

[B28] Jacobson D. R. McFarlin D. E. Kane I. Buxbaum J. N. (1992). Transthyretin Pro55, a variant associated with early-onset, aggressive, diffuse amyloidosis with cardiac and neurologic involvement. Hum. Genet. 89 (3), 353–356. 10.1007/BF00220559 1351039

[B29] Jacobson D. R. Santiago-Schwartz F. Buxbaum J. N. (1988). Restriction fragment analysis confirms the position 33 mutation in transthyretin from an Israeli patient (SKO) with familial amyloidotic polyneuropathy. Biochem. Biophys. Res. Commun. 153 (1), 198–202. 10.1016/s0006-291x(88)81208-7 2897849

[B30] Jones L. A. Skare J. C. Harding J. A. Cohen A. S. Milunsky A. Skinner M. (1991). Proline at position 36: a new transthyretin mutation associated with familial amyloidotic polyneuropathy. Am. J. Hum. Genet. 48 (5), 979–982. 1850191 PMC1683065

[B31] Karagöz G. E. Acosta-Alvear D. Walter P. (2019). The unfolded protein response: detecting and responding to fluctuations in the protein-folding capacity of the endoplasmic reticulum. Cold Spring Harb. Perspect. Biol. 11 (9), a033886. 10.1101/cshperspect.a033886 30670466 PMC6719602

[B32] Kawaguchi R. Yu J. Honda J. Hu J. Whitelegge J. Ping P. (2007). A membrane receptor for retinol binding protein mediates cellular uptake of vitamin A. Science 315 (5813), 820–825. 10.1126/science.1136244 17255476

[B33] Lamb Y. N. Deeks E. D. (2019). Tafamidis: a review in transthyretin amyloidosis with polyneuropathy. Drugs 79 (8), 863–874. 10.1007/s40265-019-01129-6 31098895

[B34] Levy J. Hawkins P. N. Rowczenio D. Godfrey T. Stawell R. Zamir E. (2012). Familial amyloid polyneuropathy associated with the novel transthyretin variant Arg34Gly. Amyloid 19 (4), 201–203. 10.3109/13506129.2012.724035 22973891

[B35] Li Z. Du K. Chu X. Lv H. Zhang W. Wang Z. (2022). *TTR* Gly83Arg mutation: beyond familial vitreous amyloidosis. Front. Neurol. 12, 821003. 10.3389/fneur.2021.821003 35185758 PMC8850374

[B36] Lim A. Prokaeva T. McComb M. E. Connors L. H. Skinner M. Costello C. E. (2003). Identification of S-sulfonation and S-thiolation of a novel transthyretin Phe33Cys variant from a patient diagnosed with familial transthyretin amyloidosis. Protein Sci. 12 (8), 1775–1785. 10.1110/ps.0349703 12876326 PMC2323963

[B37] Liu T. Zhang B. Jin X. Wang W. Lee J. Li J. (2014). Ophthalmic manifestations in a Chinese family with familial amyloid polyneuropathy due to a TTR Gly83Arg mutation. Eye 28 (1), 26–33. 10.1038/eye.2013.217 24113303 PMC3890754

[B38] Lladó L. Baliellas C. Casasnovas C. Ferrer I. Fabregat J. Ramos E. (2010). Risk of transmission of systemic transthyretin amyloidosis after domino liver transplantation. Liver Transpl. 16 (12), 1386–1392. 10.1002/lt.22174 21117248

[B39] Long D. Zeng J. Wu L. Q. Tang L. S. Wang H. L. Wang H. (2012). Vitreous amyloidosis in two large mainland Chinese kindreds resulting from transthyretin variant Lys35Thr and Leu55Arg. Ophthalmic Genet. 33 (1), 28–33. 10.3109/13816810.2011.599356 21843040

[B40] Macedo B. Batista A. R. do Amaral J. B. Saraiva M. J. (2007). Biomarkers in the assessment of therapies for familial amyloidotic polyneuropathy. Mol. Med. 13 (11-12), 584–591. 10.2119/2007-00068.Macedo 17932549 PMC2017105

[B41] Macedo B. Batista A. R. Ferreira N. Almeida M. R. Saraiva M. J. (2008). Anti-apoptotic treatment reduces transthyretin deposition in a transgenic mouse model of familial amyloidotic polyneuropathy. Biochim. Biophys. Acta 1782 (9), 517–522. 10.1016/j.bbadis.2008.05.005 18572024

[B42] Magalhães J. Eira J. Liz M. A. (2021). The role of transthyretin in cell biology: impact on human pathophysiology. Cell Mol. Life Sci. 78 (17–18), 6105–6117. 10.1007/s00018-021-03899-3 34297165 PMC11073172

[B43] Martin S. E. Benson M. D. Hattab E. M. (2014). The pathologic spectrum of oculoleptomeningeal amyloidosis with Val30Gly transthyretin gene mutation in a postmortem case. Hum. Pathol. 45 (5), 1105–1108. 10.1016/j.humpath.2013.10.037 24613567

[B44] Martin Ask N. Leung M. Radhakrishnan R. Lobo G. P. (2021). Vitamin A transporters in visual function: A mini review on membrane receptors for dietary vitamin A uptake, storage, and transport to the eye. Nutrients 13 (11), 3987. 10.3390/nu13113987 34836244 PMC8620617

[B45] Mesgarzadeh J. S. Romine I. C. Smith-Cohen E. M. Grandjean J. M. D. Kelly J. W. Genereux J. C. (2022). ATF6 activation reduces amyloidogenic transthyretin secretion through increased interactions with endoplasmic reticulum proteostasis factors. Cells 11 (10), 1661. 10.3390/cells11101661 35626697 PMC9139617

[B46] Minnella A. M. Rissotto R. Antoniazzi E. Di Girolamo M. Luigetti M. Maceroni M. (2021). Ocular involvement in hereditary amyloidosis. Genes (Basel) 12 (7), 955. 10.3390/genes12070955 34206500 PMC8304974

[B47] Monteiro C. Martins da Silva A. Ferreira N. Mesgarzadeh J. Novais M. Coelho T. (2018). Cerebrospinal fluid and vitreous body exposure to orally administered tafamidis in hereditary ATTRV30M (p.TTRV50M) amyloidosis patients. Amyloid 25 (2), 120–128. 10.1080/13506129.2018.1479249 29993288 PMC6177313

[B48] Nishimoto K. Toya Y. Davis C. R. Tanumihardjo S. A. Welham N. V. (2020). Dynamics of vitamin A uptake, storage, and utilization in vocal fold mucosa. Mol. Metab. 40, 101025. 10.1016/j.molmet.2020.101025 32473404 PMC7322172

[B49] Plate L. Wiseman R. L. (2017). Regulating secretory proteostasis through the unfolded protein response: from function to therapy. Trends Cell Biol. 27 (10), 722–737. 10.1016/j.tcb.2017.05.006 28647092 PMC5612838

[B50] Preissler S. Ron D. (2019). Early events in the endoplasmic reticulum unfolded protein response. Cold Spring Harb. Perspect. Biol. 11 (4), a033894. 10.1101/cshperspect.a033894 30396883 PMC6442202

[B51] Radhakrishnan R. Leung M. Roehrich H. Walterhouse S. Kondkar A. A. Fitzgibbon W. (2022). Mice lacking the systemic vitamin A receptor RBPR2 show decreased ocular retinoids and loss of visual function. Nutrients 14 (12), 2371. 10.3390/nu14122371 35745101 PMC9231411

[B52] Ran L. X. Zheng Z. Y. Xie B. Nie X. M. Chen X. W. Su G. (2018). A mouse model of a novel missense mutation (Gly83Arg) in a Chinese kindred manifesting vitreous amyloidosis only. Exp. Eye Res. 169, 13–19. 10.1016/j.exer.2018.01.017 29360446

[B53] Reilly M. M. Adams D. Booth D. R. Davis M. B. Said G. Laubriat-Bianchin M. (1995). Transthyretin gene analysis in European patients with suspected familial amyloid polyneuropathy. Brain 118, 849–856. 10.1093/brain/118.4.849 7655883

[B54] Reynolds M. M. Veverka K. K. Gertz M. A. Dispenzieri A. Zeldenrust S. R. Leung N. (2017). Ocular manifestations of familial transthyretin amyloidosis. Am. J. Ophthalmol. 183, 156–162. 10.1016/j.ajo.2017.09.001 28911993

[B55] Romine I. C. Wiseman R. L. (2019). PERK signaling regulates extracellular proteostasis of an amyloidogenic protein during endoplasmic reticulum stress. Sci. Rep. 9 (1), 410. 10.1038/s41598-018-37207-0 30675021 PMC6344643

[B56] Romine I. C. Wiseman R. L. (2020). Starting at the beginning: endoplasmic reticulum proteostasis and systemic amyloid disease. Biochem. J. 477 (9), 1721–1732. 10.1042/BCJ20190312 32412081

[B57] Saeki Y. Ueno S. Yorifuji S. Sugiyama Y. Ide Y. Matsuzawa Y. (1991). New mutant gene (transthyretin Arg 58) in cases with hereditary polyneuropathy detected by non-isotope method of single-strand conformation polymorphism analysis. Biochem. Biophys. Res. Commun. 180 (1), 380–385. 10.1016/s0006-291x(05)81304-x 1656975

[B58] Sanguinetti C. Minniti M. Susini V. Caponi L. Panichella G. Castiglione V. (2022). The journey of human transthyretin: synthesis, structure stability, and catabolism. Biomedicines 10 (8), 1906. 10.3390/biomedicines10081906 36009453 PMC9405911

[B59] Sato T. Susuki S. Suico M. A. Miyata M. Ando Y. Mizuguchi M. (2007). Endoplasmic reticulum quality control regulates the fate of transthyretin variants in the cell. EMBO J. 26 (10), 2501–2512. 10.1038/sj.emboj.7601685 17431395 PMC1868898

[B60] Schweitzer K. Ehmann D. Garcia R. Alport E. (2009). Oculoleptomeningeal amyloidosis in 3 individuals with the transthyretin variant Tyr69His. Can. J. Ophthalmol. 44 (3), 317–319. 10.3129/i09-023 19491989

[B61] Sekijima Y. Hammarström P. Matsumura M. Shimizu Y. Iwata M. Tokuda T. (2003). Energetic characteristics of the new transthyretin variant A25T may explain its atypical central nervous system pathology. Lab. Invest 83 (3), 409–417. 10.1097/01.lab.0000059937.11023.1f 12649341

[B62] Sekijima Y. Wiseman R. L. Matteson J. Hammarström P. Miller S. R. Sawkar A. R. (2005). The biological and chemical basis for tissue-selective amyloid disease. Cell 121 (1), 73–85. 10.1016/j.cell.2005.01.018 15820680

[B63] Shoulders M. D. Ryno L. M. Genereux J. C. Moresco J. J. Tu P. G. Wu C. (2013). Stress-independent activation of XBP1s and/or ATF6 reveals three functionally diverse ER proteostasis environments. Cell Rep. 3 (4), 1279–1292. 10.1016/j.celrep.2013.03.024 23583182 PMC3754422

[B64] Si J. B. Kim B. Kim J. H. (2021). Transthyretin misfolding, A fatal structural pathogenesis mechanism. Int. J. Mol. Sci. 22 (9), 4429. 10.3390/ijms22094429 33922648 PMC8122960

[B65] Skinner M. Harding J. Skare I. Jones L. A. Cohen A. S. Milunsky A. (1992). A new transthyretin mutation associated with amyloidotic vitreous opacities. Asparagine for isoleucine at position 84. Ophthalmology 99 (4), 503–508. 10.1016/s0161-6420(92)31949-9 1350083

[B66] Sörgjerd K. Ghafouri B. Jonsson B. H. Kelly J. W. Blond S. Y. Hammarström P. (2006). Retention of misfolded mutant transthyretin by the chaperone BiP/GRP78 mitigates amyloidogenesis. J. Mol. Biol. 356 (2), 469–482. 10.1016/j.jmb.2005.11.051 16376939

[B67] Steinhoff J. S. Lass A. Schupp M. (2022). Retinoid homeostasis and beyond: how retinol binding protein 4 contributes to health and disease. Nutrients 14 (6), 1236. 10.3390/nu14061236 35334893 PMC8951293

[B68] Sun Z. Brodsky J. L. (2019). Protein quality control in the secretory pathway. J. Cell Biol. 218 (10), 3171–3187. 10.1083/jcb.201906047 31537714 PMC6781448

[B69] Tachibana N. Tokuda T. Yoshida K. Taketomi T. Nakazato M. Li Y. F. (1999). Usefulness of MALDI/TOF mass spectrometry of immunoprecipitated serum variant transthyretin in the diagnosis of familial amyloid polyneuropathy. Amyloid 6 (4), 282–288. 10.3109/13506129909007341 10611950

[B70] Togashi S. Watanabe H. Nagasaka T. Shindo K. Shiozawa Z. Maeda S. (1999). An aggressive familial amyloidotic polyneuropathy caused by a new variant transthyretin Lys 54. Neurology 53 (3), 637–639. 10.1212/wnl.53.3.637 10449136

[B71] Uemichi T. Murrell J. R. Zeldenrust S. Benson M. D. (1992). A new mutant transthyretin (Arg 10) associated with familial amyloid polyneuropathy. J. Med. Genet. 29 (12), 888–891. 10.1136/jmg.29.12.888 1362222 PMC1016207

[B72] Uemichi T. Uitti R. J. Koeppen A. H. Donat J. R. Benson M. D. (1999). Oculoleptomeningeal amyloidosis associated with a new transthyretin variant Ser64. Arch. Neurol. 56 (9), 1152–1155. 10.1001/archneur.56.9.1152 10488818

[B73] Ueno S. Uemichi T. Yorifuji S. Tarui S. (1990). A novel variant of transthyretin (Tyr114 to Cys) deduced from the nucleotide sequences of gene fragments from familial amyloidotic polyneuropathy in Japanese sibling cases. Biochem. Biophys. Res. Commun. 169 (1), 143–147. 10.1016/0006-291x(90)91445-x 2161654

[B74] Wagner C. Hois V. Pajed L. Pusch L. M. Wolinski H. Trauner M. (2020). Lysosomal acid lipase is the major acid retinyl ester hydrolase in cultured human hepatic stellate cells but not essential for retinyl ester degradation. Biochim. Biophys. Acta Mol. Cell Biol. Lipids 1865 (8), 158730. 10.1016/j.bbalip.2020.158730 32361002 PMC7279957

[B75] White J. T. Kelly J. W. (2001). Support for the multigenic hypothesis of amyloidosis: the binding stoichiometry of retinol-binding protein, vitamin A, and thyroid hormone influences transthyretin amyloidogenicity in vitro. Proc. Natl. Acad. Sci. U. S. A. 98 (23), 13019–13024. 10.1073/pnas.241406698 11687657 PMC60817

[B76] Wiseman R. L. Mesgarzadeh J. S. Hendershot L. M. (2022). Reshaping endoplasmic reticulum quality control through the unfolded protein response. Mol. Cell 82 (8), 1477–1491. 10.1016/j.molcel.2022.03.025 35452616 PMC9038009

[B77] Wiseman R. L. Powers E. T. Buxbaum J. N. Kelly J. W. Balch W. E. (2007). An adaptable standard for protein export from the endoplasmic reticulum. Cell 131 (4), 809–821. 10.1016/j.cell.2007.10.025 18022373

[B78] Xie B. Cai S. J. Jiang M. Li H. Su G. (2017). Familial vitreous amyloidosis resulting from transthyretin variant Gly83Arg. Acta Ophthalmol. 95 (6), e520–e521. 10.1111/aos.13425 28266151

[B79] Yazaki M. Connors L. H. Eagle R. C. Jr Leff S. R. Skinner M. Benson M. D. (2002). Transthyretin amyloidosis associated with a novel variant (Trp41Leu) presenting with vitreous opacities. Amyloid 9 (4), 263–267. 10.3109/13506120209114104 12557756

[B80] Yazaki M. Varga J. Dyck P. J. Benson M. D. (2002). A new transthyretin variant Leu55Gln in a patient with systemic amyloidosis. Amyloid 9 (4), 268–271. 10.3109/13506120209114105 12557757

[B81] Yin J. Xia X. Shi Y. Lu Y. Zhao C. Huang Z. (2014). Chinese familial transthyretin amyloidosis with vitreous involvement is associated with the transthyretin mutation Gly83Arg: a case report and literature review. Amyloid 21 (2), 140–142. 10.3109/13506129.2014.892871 24601824

[B82] Zanotti G. Folli C. Cendron L. Alfieri B. Nishida S. K. Gliubich F. (2008). Structural and mutational analyses of protein-protein interactions between transthyretin and retinol-binding protein. FEBS J. 275 (23), 5841–5854. 10.1111/j.1742-4658.2008.06705.x 19021760

